# Effectiveness of Human–Artificial Intelligence Collaboration in Cephalometric Landmark Detection

**DOI:** 10.3390/jpm12030387

**Published:** 2022-03-03

**Authors:** Van Nhat Thang Le, Junhyeok Kang, Il-Seok Oh, Jae-Gon Kim, Yeon-Mi Yang, Dae-Woo Lee

**Affiliations:** 1 Department of Pediatric Dentistry and Institute of Oral Bioscience, School of Dentistry, Jeonbuk National University, Jeonju 54896, Korea; lvnthang@hueuni.edu.vn (V.N.T.L.); pedokjg@jbnu.ac.kr (J.-G.K.); pedo1997@jbnu.ac.kr (Y.-M.Y.); 2 Research Institute of Clinical Medicine, Jeonbuk National University, Jeonju 54907, Korea; 3 Biomedical Research Institute, Jeonbuk National University Hospital, Jeonju 54907, Korea; 4 Faculty of Odonto-Stomatology, Hue University of Medicine and Pharmacy, Hue University, Hue 49120, Vietnam; 5 Division of Computer Science and Engineering, Jeonbuk National University, Jeonju 54907, Korea; k1101jh@naver.com (J.K.); isoh@jbnu.ac.kr (I.-S.O.)

**Keywords:** cephalometric landmark detection, clinical application, deep learning

## Abstract

Detection of cephalometric landmarks has contributed to the analysis of malocclusion during orthodontic diagnosis. Many recent studies involving deep learning have focused on head-to-head comparisons of accuracy in landmark identification between artificial intelligence (AI) and humans. However, a human–AI collaboration for the identification of cephalometric landmarks has not been evaluated. We selected 1193 cephalograms and used them to train the deep anatomical context feature learning (DACFL) model. The number of target landmarks was 41. To evaluate the effect of human–AI collaboration on landmark detection, 10 images were extracted randomly from 100 test images. The experiment included 20 dental students as beginners in landmark localization. The outcomes were determined by measuring the mean radial error (MRE), successful detection rate (SDR), and successful classification rate (SCR). On the dataset, the DACFL model exhibited an average MRE of 1.87 ± 2.04 mm and an average SDR of 73.17% within a 2 mm threshold. Compared with the beginner group, beginner–AI collaboration improved the SDR by 5.33% within a 2 mm threshold and also improved the SCR by 8.38%. Thus, the beginner–AI collaboration was effective in the detection of cephalometric landmarks. Further studies should be performed to demonstrate the benefits of an orthodontist–AI collaboration.

## 1. Introduction

In orthodontics, detection of cephalometric landmarks refers to the localization of anatomical landmarks of the skull and surrounding soft tissues on lateral cephalograms. Since the introduction of lateral cephalograms by Broadbent and Hofrath in 1931, this approach has contributed to the analysis of malocclusion and has become a standardized diagnostic method in orthodontic practice and research [1]. In the last decade, an advanced machine-learning method called “deep learning” has received attention. Several studies have been conducted to improve the accuracy of landmark identification using lateral cephalograms. Deep learning-based reports using convolutional neural networks (CNNs) have achieved remarkable results [2,3,4,5]. These results suggest that deep learning using CNNs can assist dentists to reducing clinical problems related to orthodontic diagnosis such as tediousness, time wastage, and inconsistencies within and across orthodontists.

For the detection of anatomical landmarks, the Institute of Electrical and Electronics Engineers (IEEE) International Symposium on Biomedical Imaging (ISBI) 2015 released an open dataset for training and testing of cephalograms. Despite the limited number of annotated cephalograms, many CNN-based approaches have been proposed to solve the problem associated with the detection of anatomical landmarks. In 2017, Arik et al. introduced a framework that employed a CNN to recognize landmark appearance patterns and subsequently combined it with a statistical shape model to refine the optimal positions of all landmarks [6]. To address the restricted availability of medical imaging data for network learning with respect to the localization of anatomical landmarks, Zhang et al. proposed a two-stage task-oriented deep neural network method [7]. In addition, Urschler et al. proposed a unified framework that incorporated the image appearance information as well as geometric landmark configuration into a unified random forest framework, which was optimized iteratively to refine joint landmark predictions using a coordinate descent algorithm [8]. Recently, Oh et al. proposed the deep anatomical context feature learning (DACFL) model, which employs a Laplace heatmap regression method based on a fully convolutional network. Its main mechanism is accomplished using two main schemes: local feature perturbation (LFP) and anatomical context (AC) loss. LFP can be considered a data augmentation method based on prior anatomical knowledge. It perturbs the local pattern of the cephalogram, forcing the network to seek relevant features globally. AC loss can result in a large cost when the predicted anatomical configuration of landmarks differs from the ground-truth configuration. The anatomical configuration considers the angles and distances between all landmarks. Since the proposed system follows an end-to-end learning method, only a single feed-forward execution is required in the test phase to localize all landmarks [2].

Most research to date has focused on head-to-head comparisons between artificial intelligence (AI)-based systems and dentists for the localization of cephalometric landmarks [2,3,4,5,6,9,10,11,12,13,14,15,16]. Previous studies have shown that AI is equivalent or even superior to experienced orthodontists under experimental conditions [11,13]. Rapid developments in AI-based diagnosis have made it imperative to consider the opportunities and risks of new diagnostic paradigms. In fact, competition between humans and AI is against the nature and purpose of AI. Therefore, AI support for human diagnosis may be more useful and practical. The competitive view about AI is evolving based on studies indicating that a human–AI collaboration approach is more promising. The impact of human–AI collaboration on the accuracy of cephalometric landmark detection has not been evaluated to date. This leads to the following question: can a human–AI collaboration perform better than humans or AI alone in cephalometric landmark detection?

Among the previous CNN models, DACFL outperformed other state-of-the-art methods and achieved high performance in landmark identification on the IEEE ISBI 2015 dataset [2]. Therefore, our study aimed to evaluate the effect of DACFL-based support on the clinical skills of beginners in cephalometric diagnosis. Furthermore, we used a private dataset to evaluate the performance of the DACFL model in clinical applications.

## 2. Materials and Methods

### 2.1. Data Preparation

Altogether, 1293 lateral cephalograms were collected from the Picture-Aided Communication System server (INFINITT Healthcare Co., Ltd., Seoul, Korea) at Jeonbuk National University Dental Hospital, South Korea. Furthermore, images were collected from children and adolescents aged 6–18 years who visited the Department of Pediatric Dentistry for orthodontic treatment between 2008 and 2018. All applicable data protection laws were respected. During images collection, patient information was removed from the cephalograms. This study was approved by the Institutional Review Board of Jeonbuk National University Hospital (No. CUH2019-05-057).

Lateral cephalograms were acquired for diagnostic purposes and exported in the JPG format, with resolutions varying between 550 × 550 and 4066 × 4345 pixels. The dataset was built without any restrictions in terms of sex, craniofacial or dental surgery, and treatment. In the first step, 1193 images were randomly selected as training data and 100 images were used as test data. The characteristics of data are listed in Table 1.

Data are expressed mean ± standard deviation (SD) for age and N (%) for gender, class I, II division 1, II division 2, and III.

**Figure 1:**
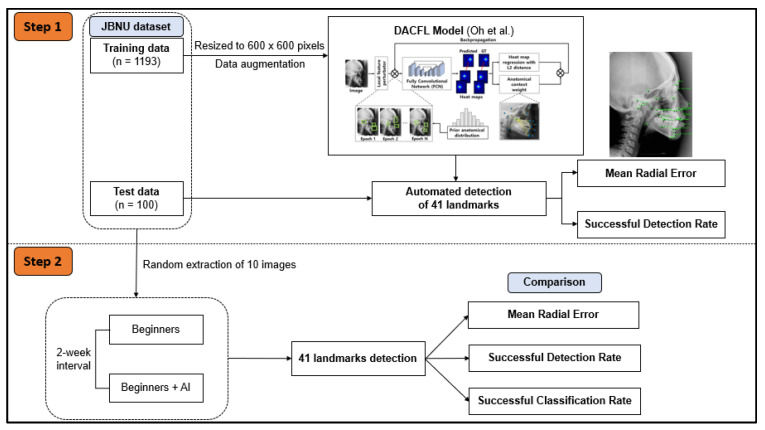
Workflow diagram of the study plan. In step 1, JBNU dataset including 1193 images for training and 100 images for testing was used to evaluate the performance of the DACFL model in clinical applications. In step 2, 10 images were extracted randomly from JBNU test data to evaluate the effect of DACFL-based support on the clinical skills of beginners in cephalometric diagnosis. Abbreviations: AI, artificial intelligence; DACFL, deep anatomical context feature learning; JBNU, Jeonbuk National University.

In the subsequent step, 10 images from the test data were extracted randomly to evaluate the impact of human–AI collaboration on the detection of cephalometric landmarks (Figure 1).

### 2.2. Manual Identification of Cephalometric Landmarks

Altogether, 41 landmarks were manually identified by dental residents at the Department of Pediatric Dentistry, Jeonbuk National University Dental Hospital, South Korea (Supplementary Table S1). A modified version of a commercial cephalometric analysis software (V-Ceph version 7, Osstem Implant Co., Ltd., Seoul, Korea) was used to digitize the records of the 41 cephalometric landmarks. This software displayed the cephalograms and obtained the coordinates of each landmark.

In this experiment, 20 final-year students from the School of Dentistry, Jeonbuk National University, South Korea were selected as beginners. Ten cephalograms that had been analyzed twice (at a 1-week interval) were used to evaluate the support ability of AI. Analyses of cephalograms were performed at the following two timepoints.

(1) Twenty dental students were educated regarding the definitions of cephalometric landmarks and the use of the V-Ceph software before the experiment. All students traced the positions of anatomical landmarks without AI support. After tracing, the ground truth was not provided for the students.
(2) After 1 week, all students traced the anatomical landmarks on 10 randomly arranged images while going through the answers provided by the AI model. The students were not reported about reusing the images from the previous experiment. These answers were displayed separately from the actual screen of landmarks. If the students changed their answers by replacing them with the answers provided by AI, the changes were recorded.

### 2.3. Network Architecture and Implementation Details

Our architecture was based on the attention U-Net [17]. The contracting path and the expansive path consisted of repeated applications of two 3 × 3 convolutions, each followed by LeakyReLU activation and 2 × 2 max pooling (for the contracting path) or up-sampling (for the expansive path) [18]. The number of feature channels increased from 64 to 128, 256, and 512 in the contracting path and decreased from 512 to 256, 128, and 64 in the expansive path. We used AC loss [2] as a cost function and minimized it by using an Adam optimizer. The initial learning rate was 1 × 10^−4^, and the learning rate was set by cosine annealing schedule.

Additionally, we performed data augmentation by rotating the input images randomly by [−25, 25], rescaling by [0.9, 1.1], and translating by [0, 0.05] for both the *x*-axis and *y*-axis. We changed the brightness, contrast, and hue of the input images randomly in the ranges [−1, 1], [−1, 1], and [−0.5, 0.5], respectively. The ranges are the ones given by PyTorch. With a 1/10 probability, we did not apply a data augmentation procedure to the input images to ensure that the deep learning model could learn the original image [2].

We trained and tested the network using an Intel Xeon Gold 6126 2.6 GHz CPU with 64 GB memory and a RTX 2080 Ti GPU with an 11 GB RAM. The average size of the input images was 2067 × 1675 pixels, and each image had a different pixel size. Therefore, we calculated the pixel length of a 50 mm X-ray ruler to calculate the pixel size for each test image. We resized the input images to 800 × 600 pixels with a mini-batch size of two to reduce the computing time. In the test phase, we resized the result to the original input size to obtain the correct result.

### 2.4. Evaluation Matrices

We used different measurement methods to measure the performance of the landmark-detection model. The positions of the landmarks were identified using the x- and y-coordinates. The mean radial error (MRE) and standard deviation (SD) are calculated as follows:

$$\mathrm{MRE}=\frac{\Sigma_{i=1}^{N}R_{i}}{N}$$

$$\mathrm{SD}=\sqrt{\frac{\Sigma_{i=1}^{N}{\left(R_{i}-MRE\right)}^{2}}{N-1}}$$

In these equations, *N* denotes the set size. The radial error (*R*) is the Euclidean distance and is defined as the distance between the predicted and actual coordinates.

The successful detection rate (SDR) is an important measure for this problem. The estimated coordinates are considered correct if the error between the estimated coordinates and the correct position is less than a precision range. The SDR was calculated as follows:

$$\mathrm{SDR}=\frac{\mathrm{number}\;\mathrm{of}\;\mathrm{successfully}\;\mathrm{detected}\;\mathrm{landmarks}\;\mathrm{with}\;\mathrm{respect}\;\mathrm{to}\;z}{N}\times 100\%$$

In this equation, z means the precision ranges of 2, 2.5, 3, and 4 mm.

For the classification of anatomical types, the eight clinical measurements set in the IEEE ISBI 2015 challenge were analyzed (Supplementary Table S2) [19,20]. The measurement values and classification results derived by the dental residents were set as the reference values, while the classification results by the AI, beginners, and beginner–AI collaboration were compared using the successful classification rate (SCR).

### 2.5. Statistical Analysis

The benefits of the beginner–AI collaboration were analyzed for each landmark. A *t*-test was applied to compare the average SDR between the beginner-only and beginner–AI groups within 2, 2.5, 3, and 4 mm thresholds. All data were analyzed using IBM SPSS Statistics (version 20; IBM Corp., Armonk, NY, USA) and PRISM (version 8.0.2; GraphPad Software, Inc.; San Diego, CA, USA). Statistical significance was set at a *p*-value < 0.05.

## 3. Results

### 3.1. Performance of the DACFL Model on the Private Dataset

#### 3.1.1. Mean Radial Error

The DACFL model showed an average MRE of 1.87 ± 2.04 mm (Table 2). Among the 41 landmarks, the sella exhibited the lowest MRE (0.76 ± 0.44 mm), while the glabella exhibited the highest MRE (5.18 ± 5.13 mm).

#### 3.1.2. Successful Detection Rate

The model achieved average SDRs of 73.32%, 80.39%, 85.61%, and 91.68% within 2, 2.5, 3, and 4 mm thresholds, respectively. Across all ranges, the sella exhibited the highest SDR, while the glabella exhibited the lowest SDR. In addition, the SDRs of maxilla 1 root (38%), mandible 6 root (36%), glabella (32%), and soft tissue nasion (38%) were low within the 2 mm threshold (Table 3).

### 3.2. Impact of Artificial Intelligence-Based Assistance on the Performance of Beginners in the Detection of Cephalometric Landmarks

#### 3.2.1. Mean Radial Error and Successful Detection Rate

**Figure 2:**
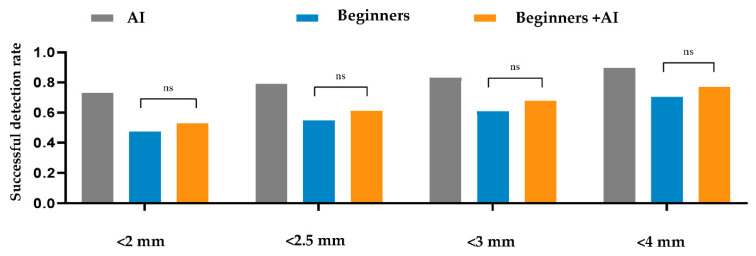
Comparison between the beginner-only and beginner–artificial intelligence groups in terms of the successful detection rate. A *t*-test was applied to compare the average successful detection rates between the beginner-only and beginner–AI groups within 2, 2.5, 3, and 4 mm thresholds. The beginner–AI collaboration improved the successful detection rates within 2, 2.5, 3, and 4 mm thresholds. Abbreviations: AI, artificial intelligence; ns, not significant.

Within a 2 mm threshold, the AI, beginner–AI, and beginner-only groups achieved SDRs of 73.17%, 52.73%, and 47.4%, respectively. Furthermore, the average MREs and SDs of AI, beginner–AI, and beginner-only groups were 1.89 ± 2.63 mm, 3.14 ± 4.06 mm, and 3.72 ± 4.52 mm, respectively. Details are reported in Table 4. Furthermore, a comparison between beginner-only and beginner–AI groups in terms of the SDR is shown in Figure 2.

**Figure 3:**
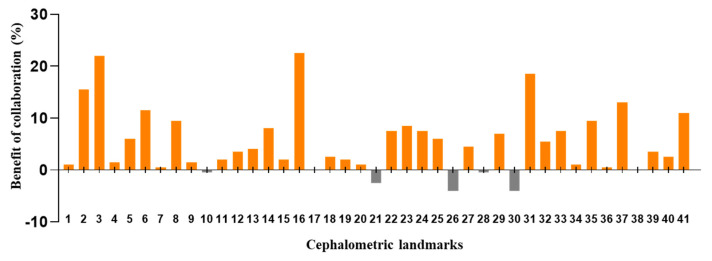
Benefit of beginner–AI collaboration in the detection of cephalometric landmarks. Based on successful detection rate for each landmark within a 2 mm threshold, the benefits of beginner–AI collaboration were analyzed. In general, this collaboration showed a positive impact on the majority of cephalometric landmarks.

**Figure 4:**
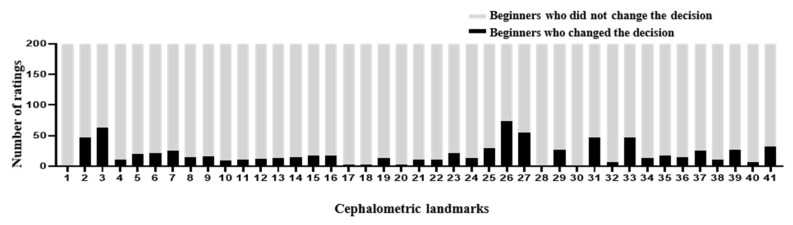
Number of decision changes among beginners across 41 landmarks. In the second experiment, the beginners traced the anatomical landmarks on 10 images with the AI’s answer view. The recorded changes are represented as number of ratings. In general, the number of decision changes was small despite being shown at most anatomical landmarks.

The DACFL model showed that the lower lip exhibited the lowest MRE (0.62 ± 0.35 mm) and the highest SDR (100%) while the glabella exhibited the highest MRE (5.72 ± 3.54 mm) and lowest SDR (20%) (Table 5). In the beginner-only group, mandible 1 crown exhibited the lowest MRE (1.31 ± 2.99 mm) and highest SDR (93%), while the glabella exhibited the highest MRE (8.9 ± 7.05 mm) and lowest SDR (20%). In the beginner–AI group, mandible 1 crown exhibited the lowest MRE (1.23 ± 2.96 mm) and highest SDR (94%), while the glabella exhibited the highest MRE (7.31 ± 5.42 mm) and lowest SDR (16%). The benefits of AI– beginner collaboration in the localization of anatomical landmarks and the number of decision changes among the beginners across 41 landmarks are presented in Figure 3 and Figure 4.

#### 3.2.2. Successful Classification Rate

**Figure 5:**
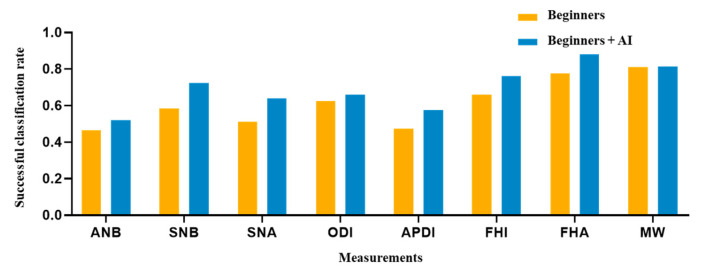
Comparison of eight clinical measurements between the beginner-only and beginner–AI groups. From the SCRs of two groups, a figure was presented to demonstrate the AI’s support. As a result, the beginner–AI collaboration improved the SCRs of eight clinical measurements. Abbreviation: SCR, successful classification rate.

The AI, beginner–AI, and beginner-only groups achieved SCRs of 83.75%, 69.69%, and 61.31%, respectively (Table 6). In the AI group, the SNA (100%) and FHA (100%) exhibited the highest SCR, while the ANB (60%) exhibited the lowest SCR. In the beginner-only group, the MW (81%) exhibited the highest SCR, while the ANB (47%) exhibited the lowest SCR. Among beginner–AI group, the FHA (88%) exhibited the highest SCR while ANB (52%) exhibited the lowest SCR. A comparison between beginner-only and beginner–AI groups in terms of eight measurement parameters is shown in Figure 5.

## 4. Discussion

### 4.1. Performance of the DACFL Model on the Private Dataset

Most previous studies have tested the accuracy of anatomical landmark detection on IEEE ISBI 2015 lateral cephalograms [2,3,4,5,6,9,15], possibly showing high comparability, but limited generalizability. Therefore, testing broad data can demonstrate the generalizability and robustness of the model. Among the previous models, the DACFL model showed a high SDR as a state-of-the-art model for cephalometric landmark detection [2]. In the case of private cephalograms, the model showed a slight reduction in the SDR within a 2 mm threshold. This result was superior or similar to those from previous studies [3,4,12]. In a previous study, an even more dramatic drop in the accuracy was observed when the models were tested on a fully external dataset [3,4]. Overall, the results for the private dataset were inferior to those for the public dataset with standardized images [2,3,4,5,6,9,15].

In the present study, the private dataset was associated with difficulties in landmark detection in children. These difficulties were probably due to low bone density, size and shape variability of anatomical structures, and the existence of primary teeth and permanent tooth germs. In addition to maxillofacial anatomy, patients’ heads vary in shape. Although we selected a reference cephalogram that was closely matched to the one from training data for each test, there were still missed situations. Correct head positioning of the patient during the procedure is important to avoid errors in the identification and measurement of landmarks [4,21,22]. It is difficult to maintain the heads of children in standard positions. In addition to the quality of dataset, the number of images and cephalometric landmarks also influence the results. A previous study showed that the accuracy of AI increased linearly with an increasing number of learning datasets and decreased with an increasing number of detection targets [23]. Our study used an insufficiently large number of images and detected 41 anatomical landmarks. The training data should be increased to an optimal number of images between 2300 and 5400 to improve the performance of landmark detection.

For clinical applications, a mean error within a 2 mm threshold has been suggested to be acceptable in many related studies [2,3,4,5,6,9,10,11,13,14,15,16,24]. Therefore, the MRE in the present study was clinically acceptable. However, while assessing which specific landmarks were prone to incorrect detection, the maxilla 1 root, mandible 6 root, glabella, and soft tissue nasion showed greater deviations. These findings are not consistent with those from previous studies [3,4,12]. This discrepancy can be explained by the fact that maxilla 1 root was affected by the existence of maxillary anterior tooth germs. The location of the apex was based on general knowledge of the expected taper perceived from the crown and visible portion of the root. This problem was also encountered during previous research on reliability [10,11,25,26,27,28]. Furthermore, the mandible 6 root was affected by overlapping structures, which is a common problem in the lateral cephalograms. Dental landmarks usually tend to have poorer validity than skeletal landmarks [10,11,27]. Soft tissue nasion and the glabella were located in areas with considerably higher dark. Thus, it was difficult to identify these soft tissue landmarks precisely, even in a magnified view.

### 4.2. Impact of AI-Based Assistance on the Performance of Beginners in Cephalometric Landmark Detection

The AI group had the highest average SDR, followed by the beginner–AI and beginner-only groups. With AI support, the average SDR increased by up to 5.33% within a 2 mm threshold, while the average MRE decreased. Detection of porion, basion, nasion, articulare, soft tissue A, soft tissue pogonion, and PPOcc improved over 10% in terms of SDR. The remaining landmarks were detected with little or no improvement in the SDR (Figure 2). In general, AI aids beginners in improving landmark detection. This was demonstrated by the impact of the beginner–AI collaboration on each landmark (Figure 3). However, the improvement was insignificant, since there were small changes in the positions of the landmarks (Figure 4).

In addition to the SDR, we calculated the SCR to evaluate the classification performance. The DACFL model showed better results than previous models [6,12,29]. As expected, measurements consisting of landmarks with higher SDRs yielded higher SCR values. The average SCRs of the three groups showed a descending trend similar to that observed in case of average SDRs (highest in the AI group, followed by the beginner–AI group and the beginner-only group). With AI support, the average SCR increased by 8.38%, but the increase was not statistically significant. This may be explained by the low increase in the SDRs with AI support. The SCRs for the measurement of SNA, SNB, APDI, FHI, and FHA improved over 10%, while the SCR showed little improvement for the remaining measurements (Table 6).

In the present study, beginners were the final-year dental students with little experience in the detection of cephalometric landmarks. The precision of landmark identification can be affected by various factors such as the level of knowledge, individual understanding of the definitions of landmarks, and quality of cephalometric images [30,31]. Among the soft tissue landmarks, glabella, soft tissue nasion, columella, soft tissue A, and stms showed low SDRs due to higher dark in these regions. Problems with image quality influenced the ability of dental students who lacked experience in cephalometric landmark detection. In a previous study, dental students showed a high variability in landmark identification results [32]. This finding is consistent with the results of the present study (Table 5). Furthermore, inexperienced annotators exhibited a lower accuracy of landmark detection than AI for lateral cephalograms, which was consistent with the results of a previous study involving frontal cephalograms [33].

Our study has several limitations. The private dataset was small and had fewer variations. The patients were children and adolescents. This might have influenced the detection of cephalometric landmark. Thus, private datasets for adults should be investigated to confirm the performance of the DACFL model. The number of cephalometric landmarks was not sufficiently large to examine the full ability the of model. Moreover, landmark identification was performed by beginners. A previous study showed that experienced orthodontists exhibited lower variability in landmark detection than dental students. Further studies are necessary to demonstrate the benefits of a collaboration between AI and experienced orthodontists.

## 5. Conclusions

Our study showed that the DACFL model achieved an SDR of 73.17% within a 2 mm threshold on a private dataset. Furthermore, the beginner–AI collaboration improved the SDR by 5.33% within a 2 mm threshold and also improved the SCR by 8.38% when compared with beginners. These results suggest that the DACFL model is applicable to clinical orthodontic diagnosis. Further studies should be performed to demonstrate the benefits of a collaboration between AI and experienced orthodontists.

## Figures and Tables

**Table 1:** Descriptive summary of study data.

Variables	Mean ± SD/N (%)
Training data	
Age (years)	9.31 ± 2.77
Gender (male)	566 (47.44)
Angle classification	
Class I	324 (27.16)
Class II division 1	291 (24.39)
Class II division 2	139 (11.65)
Class III	439 (36.80)
Test data	
Age (years)	9.74 ± 3.12
Gender (male)	47 (47.00)
Angle classification	
Class I	27 (27.00)
Class II division 1	24 (24.00)
Class II division 2	12 (12.00)
Class III	37 (37.00)

**Table 2:** Results of landmark detection in terms of mean radial error.

No.	Landmarks	AI	
		MRE (mm)	SD
1	Sella	0.76	0.44
2	Porion	1.40	1.20
3	Basion	2.09	1.98
4	Hinge axis	1.70	1.06
5	Pterygoid	2.38	1.46
6	Nasion	1.33	0.87
7	Orbitale	2.23	1.81
8	A-point	1.43	1.11
9	PM	1.32	0.88
10	Pogonion	1.19	0.96
11	B-point	1.69	1.22
12	Posterior nasal spine	1.63	1.37
13	Anterior nasal spine	1.27	0.91
14	R1	2.32	1.62
15	R3	1.84	1.21
16	Articulare	1.03	0.74
17	Menton	1.22	0.91
18	Maxilla 1 crown	1.03	0.92
19	Maxilla 1 root	3.31	2.78
20	Mandible 1 crown	0.90	0.52
21	Mandible 1 root	2.89	4.04
22	Maxilla 6 distal	1.41	1.86
23	Maxilla 6 root	2.20	1.53
24	Mandible 6 distal	1.64	2.32
25	Mandible 6 root	2.97	2.50
26	Glabella	5.18	5.13
27	Soft tissue nasion	3.10	2.44
28	Pronasale	2.06	8.16
29	Columella	1.05	0.87
30	Subnasale	1.06	0.99
31	Soft tissue A	1.21	1.34
32	Upper lip	1.48	4.10
33	Stms	1.82	1.46
34	Stmi	1.03	0.79
35	Lower lip	1.16	0.90
36	Soft tissue B	2.08	2.70
37	Soft tissue pogonion	4.70	10.39
38	Gnathion	1.34	2.08
39	Gonion	2.70	2.14
40	APOcc	1.02	1.19
41	PPOcc	2.33	2.86
Average		1.87	2.04

Abbreviations: AI, artificial intelligence; MRE, mean radial error; SD, standard deviation.

**Table 3:** Results of landmark detection in terms of successful detection rate.

No.	Landmarks	SDR (%)			
		<2 mm	<2.5 mm	<3 mm	<4 mm
1	Sella	97%	100%	100%	100%
2	Porion	84%	92%	94%	95%
3	Basion	59%	73%	82%	89%
4	Hinge axis	64%	79%	90%	97%
5	Pterygoid	48%	58%	69%	85%
6	Nasion	83%	92%	93%	99%
7	Orbitale	59%	70%	79%	90%
8	A-point	82%	89%	91%	96%
9	PM	80%	88%	95%	100%
10	Pogonion	86%	91%	95%	97%
11	B-point	69%	80%	84%	91%
12	Posterior nasal spine	74%	86%	89%	97%
13	Anterior nasal spine	85%	92%	92%	99%
14	R1	51%	59%	73%	85%
15	R3	61%	77%	88%	94%
16	Articulare	90%	95%	97%	99%
17	Menton	86%	89%	96%	96%
18	Maxilla 1 crown	91%	94%	94%	97%
19	Maxilla 1 root	38%	49%	60%	73%
20	Mandible 1 crown	96%	98%	100%	100%
21	Mandible 1 root	48%	58%	70%	82%
22	Maxilla 6 distal	87%	92%	95%	98%
23	Maxilla 6 root	56%	68%	78%	93%
24	Mandible 6 distal	84%	88%	88%	92%
25	Mandible 6 root	36%	53%	68%	80%
26	Glabella	32%	40%	46%	58%
27	Soft tissue nasion	38%	47%	60%	76%
28	Pronasale	95%	95%	95%	95%
29	Columella	96%	96%	97%	97%
30	Subnasale	93%	96%	97%	98%
31	Soft tissue A	89%	93%	94%	97%
32	Upper lip	89%	91%	93%	97%
33	Stms	68%	77%	83%	92%
34	Stmi	87%	93%	96%	100%
35	Lower lip	86%	88%	92%	99%
36	Soft tissue B	74%	81%	82%	87%
37	Soft tissue pogonion	62%	69%	76%	80%
38	Gnathion	90%	92%	95%	96%
39	Gonion	51%	56%	68%	79%
40	APOcc	93%	94%	95%	98%
41	PPOcc	69%	78%	81%	86%
Average		73.32%	80.39%	85.61%	91.68%

Abbreviation: SDR, successful detection rate.

**Table 4:** Quantitative comparison by average successful detection rate and mean radial error.

Group	SDR (%)				MRE (mm)	SD
	<2 mm	<2.5 mm	<3 mm	<4 mm		
AI	73.17%	79.02%	83.17%	89.51%	1.89	2.63
Beginners	47.40%	54.83%	60.80%	70.21%	3.72	4.52
Beginners + AI	52.73%	61.16%	67.77%	77.01%	3.14	4.06

**Table 5:** Successful detection rate and mean radial error for each landmark within a 2 mm threshold.

No.	Landmarks	AI		Beginners		Beginners + AI	
		SDR (%)	MRE ± SD (mm)	SDR (%)	MRE ± SD (mm)	SDR (%)	MRE ± SD (mm)
1	Sella	90%	1.14 ± 0.52	83.5%	1.67 ± 2.36	84.5%	1.74 ± 2.42
2	Porion	90%	1.19 ± 0.6	25.5%	5.3 ± 4.22	41%	3.34 ± 3.25
3	Basion	70%	2.21 ± 1.88	22%	6.3 ± 4.92	44%	3.58 ± 3.3
4	Hinge axis	70%	1.77 ± 1.35	54.5%	2.56 ± 2.38	56%	2.47 ± 2.44
5	Pterygoid	70%	2.12 ± 1.56	41.5%	3.71 ± 3.11	47.5%	3.27 ± 2.88
6	Nasion	70%	1.62 ± 0.72	42.5%	5.06 ± 5.73	54%	3.25 ± 4.51
7	Orbitale	40%	3.01 ± 2.46	21.5%	4.66 ± 3.4	22%	4.26 ± 3.02
8	A-point	70%	1.8 ± 0.98	45.5%	3.21 ± 3.13	55%	2.74 ± 3.05
9	PM	70%	1.89 ± 0.92	48%	3.06 ± 3.53	49.5%	2.88 ± 3.41
10	Pogonion	90%	1.33 ± 1.06	64%	2.51 ± 3.61	63.5%	2.35 ± 3.41
11	B-point	70%	1.76 ± 0.86	43.5%	3.27 ± 3.6	45.5%	3.1 ± 3.4
12	Posterior nasal spine	70%	1.5 ± 1.02	36.5%	3.77 ± 4.21	40%	3.45 ± 4.16
13	Anterior nasal spine	80%	1.29 ± 0.76	57.5%	2.79 ± 3.92	61.5%	2.59 ± 3.88
14	R1	60%	2.02 ± 1.32	28%	4.05 ± 3.85	36%	3.49 ± 2.74
15	R3	70%	1.74 ± 1.53	36%	3.82 ± 3.55	38%	3.44 ± 3.35
16	Articulare	100%	0.88 ± 0.45	58.5%	2.51 ± 2.62	81%	1.73 ± 2.41
17	Menton	90%	1.26 ± 0.74	67%	2.26 ± 3.15	67%	2.28 ± 3.14
18	Maxilla 1 crown	90%	1.1 ± 0.57	86%	1.48 ± 3.14	88.5%	1.41 ± 3.16
19	Maxilla 1 root	50%	2.9 ± 1.66	32%	3.71 ± 2.96	34%	3.41 ± 3.02
20	Mandible 1 crown	90%	0.99 ± 0.58	92.5%	1.31 ± 2.99	93.5%	1.23 ± 2.96
21	Mandible 1 root	70%	1.91 ± 1.52	42%	3.2 ± 3.21	39.5%	3.12 ± 3.06
22	Maxilla 6 distal	100%	0.93 ± 0.44	73%	2.76 ± 3.46	80.5%	2.11 ± 2.9
23	Maxilla 6 root	50%	2.13 ± 1.27	42%	3.16 ± 2.8	50.5%	2.62 ± 2.46
24	Mandible 6 distal	100%	0.93 ± 0.6	67.5%	3.1 ± 4.24	75%	2.29 ± 3.46
25	Mandible 6 root	20%	3.38 ± 1.97	29%	4.53 ± 4.29	35%	3.49 ± 3.47
26	Glabella	20%	5.72 ± 3.54	19.5%	8.9 ± 7.05	15.5%	7.31 ± 5.42
27	Soft tissue nasion	30%	3.7 ± 2.95	18.5%	6.29 ± 5.19	23%	4.93 ± 4.26
28	Pronasale	90%	4.67 ± 11.98	82.5%	5.24 ± 11.91	82%	5.29 ± 11.93
29	Columella	90%	1.27 ± 1.54	36.5%	3.47 ± 3.42	43.5%	3.04 ± 3.28
30	Subnasale	100%	0.86 ± 0.43	83.5%	1.79 ± 2.86	79.5%	1.84 ± 2.85
31	Soft tissue A	70%	1.37 ± 0.93	31.5%	4.02 ± 3.68	50%	2.66 ± 3.07
32	Upper lip	90%	1.68 ± 2.23	53%	3.12 ± 4.02	58.5%	2.89 ± 3.98
33	Stms	50%	2.3 ±1.91	0%	7.11 ± 3.02	7.5%	6.38 ± 3.2
34	Stmi	90%	0.8 ± 0.57	63%	2.51 ± 3.52	64%	2.39 ± 3.49
35	Lower lip	100%	0.62 ± 0.35	65.5%	2.45 ± 3.49	75%	2.01 ± 3.36
36	Soft tissue B	60%	2.39 ± 1.87	45%	3.05 ± 3.54	45.5%	2.93 ± 3.33
37	Soft tissue pogonion	70%	1.22 ± 0.94	41%	4.61 ± 4.86	54%	3.14 ± 3.94
38	Gnathion	100%	0.7 ± 0.52	66.5%	2.16 ± 3.15	66.5%	2.12 ± 3.13
39	Gonion	50%	2.92 ± 1.74	23%	4.23 ± 3.07	26.5%	3.92 ± 2.89
40	APOcc	90%	1.83 ± 2.76	69%	2.8 ± 4.07	71.5%	2.75 ± 4.03
41	PPOcc	60%	2.53 ± 2.91	6%	7.09 ± 5.56	17%	5.59 ± 5.04

**Table 6:** Successful classification rate for eight clinical measurements.

Measurements	SCR (%)		
	AI	Beginners	Beginners + AI
ANB	60%	46.5%	52%
SNB	90%	58.5%	72.5%
SNA	100%	51%	64%
ODI	70%	62.5%	66%
APDI	90%	47.5%	57.5%
FHI	90%	66%	76%
FHA	100%	77.5%	88%
MW	70%	81%	81.5%
Average	83.75%	61.31%	69.69%

## Data Availability

The datasets used and/or analyzed during the present study can be obtained from the corresponding author upon reasonable request.

## Supplementary Materials

The following supporting information can be downloaded at: https://www.mdpi.com/article/10.3390/jpm12030387/s1, Table S1, List of anatomical landmarks; Table S2, Classification criteria for anatomical types.
