# Supplementary material for: Effectiveness of Human–Artificial Intelligence Collaboration in Cephalometric Landmark Detection
Source: J Pers Med. 2022 Mar 3;12(3):387. doi: 10.3390/jpm12030387 (PMC8954049; doi:10.3390/jpm12030387)
Supplement: Supplementary file 1 [file jpm-12-00387-s001.zip › Supplementary Table S1.pdf]

Journal of *Personalized Medicine*

MDPI

Table S1. List of anatomical landmarks.

No.	Landmarks	Definition
1	Sella	Midpoint of the sella turcica or the hypophyseal/pituitary fossa
2	Porion	Highest point on the roof of the left external auditory meatus
3	Basion	Point at the center of the anterior border of foramen magnum at the base of the occipital bone
4	Hinge axis	Center of rotation of the condyle
5	Pterygoid	Eleven o'clock position from the pterygomaxillary fissure
6	Nasion	Most anterior point on the frontonasal suture in the middle
7	Orbitale	Lowest point on the inferior margin of the orbit, midpoint between the right and left images
8	A-point	Deepest point on the curved bony outline between the anterior nasal spine and prosthion
9	PM	Point selected on the anterior border of the symphysis where the curvature changes between B-point and pogonion
10	Pogonion	Most anterior point on the symphysis of the mandible
11	B-point	Deepest midline point on the mandible between infradentale and pogonion
12	Posterior nasal spine	Intersection of the continuation of the anterior wall of the pterygopalatine fossa and the nasal floor
13	Anterior nasal spine	Tip of the bony anterior nasal spine in the midline or median plane
14	R1	Deepest point on the curve of the anterior border of the ramus
15	R3	Most inferior point on the sigmoid notch of the ramus
16	Articulare	Intersection of the dorsal contours of the processus articularis mandibulare and os temporale
17	Menton	Lowermost point on the chin contour
18	Maxilla 1 crown	Tip of the maxillary incisor crown
19	Maxilla 1 root	Tip of the maxillary incisor root
20	Mandible 1 crown	Tip of the mandibular incisor crown
21	Mandible 1 root	Tip of the mandibular incisor root
22	Maxilla 6 distal	Distal contact point of the maxillary first molar
23	Maxilla 6 root	Distobuccal root of the maxillary first molar
24	Mandible 6 distal	Distal contact point of the mandibular first molar
25	Mandible 6 root	Distal root of the mandibular first molar
26	Glabella	Most prominent or anterior point in the midsagittal plane of the forehead at the level of the superior orbital ridges
27	Soft tissue nasion	Most concave or retruded point in the tissue overlying the area of the frontonasal suture

28	Pronasale	Most prominent or anterior point on the tip of the nose
29	Columella	Most anterior point on the columella of the nose
30	Subnasale	Point at which the nasal septum between the nostrils merges with the upper cutaneous lip in the midsagittal plane
31	Soft tissue A	Point of greatest concavity in the midline of the upper lip between subnasale and labrale superius
32	Upper lip	Most anterior point on the margin of the upper membranous lip
33	Stms	Lowermost midline point on the vermilion of the upper lip
34	Stmi	Uppermost midline point on the vermilion of the lower lip
35	Lower lip	Most anterior point on the margin of the lower membranous lip
36	Soft tissue B	Point of greatest concavity in the midline of the lower lip between labrale inferius and soft tissue pogonion
37	Soft tissue pogonion	Most prominent or anterior point on the soft tissue chin in the midsagittal plane
38	Gnathion	Most anterior-inferior point on the lateral shadow of the chin (usually best determined by selecting the midpoint between pogonion and menton on the contour of the chin)
39	Gonion	Mediolateral midpoint on the posteriormost border of each gonial angle (gonion is a bilateral structure)
40	APOcc	Midpoint of the incisor overbite in occlusion
41	PPOcc	Most distal point of contact between the most posterior molars in occlusion
