# Supplementary material for: Effectiveness of Human–Artificial Intelligence Collaboration in Cephalometric Landmark Detection
Source: J Pers Med. 2022 Mar 3;12(3):387. doi: 10.3390/jpm12030387 (PMC8954049; doi:10.3390/jpm12030387)
Supplement: Supplementary file 1 [file jpm-12-00387-s001.zip › Supplementary Table S2.pdf]

Journal of
*Personalized
Medicine*

Table S2. Classification criteria for anatomical types.

Variables	Type 1	Type 2	Type 3	Type 4
ANB	3.2–5.7°<br>Class I, normal	> 5.7°<br>Class II	< 3.2°<br>Class III	
SNA	79.4–83.2°<br>Normal maxilla	> 83.2°<br>Prognathic maxilla	< 79.4°<br>Retrognathic maxilla	
SNB	74.6–78.7°<br>Normal mandible	< 74.6°<br>Retrognathic mandible	> 78.7°<br>Prognathic mandible	
ODI	74.5 ± 6.1°	> 80.5°<br>Deep bite tendency	< 68.4°<br>Open bite tendency	
APDI	81.4 ± 3.8°	< 77.6°<br>Class II tendency	> 85.2°<br>Class III tendency	
FHI	0.65–0.75	> 0.75<br>Short face tendency	< 0.65<br>Long face tendency	
FHA	26.8–31.4°	> 31.4°<br>High mandibular angle	< 26.8°<br>Low mandibular angle	
MW	2–4.5 mm	0 mm<br>Edge-to-edge	< 0 mm<br>Anterior cross bite	> 4.5 mm<br>Large overjet

Abbreviations:

ANB, angle between A-point, nasion and B-point

SNA, angle between sella, nasion and A-point

SNB, angle between sella, nasion and B-point

Overbite depth indicator (ODI), sum of the angle between the lines from A-point to B-point and from menton to gonion, and the angle between the lines from orbitale to porion and from posterior nasal spine to anterior nasal spine

Anteroposterior dysplasia indicator (APDI), sum of the angle between the lines from orbitale to porion and from nasion to pogonion, the angle between the lines from nasion to pogonion and from A-point to B-point, and the angle between the lines from orbitale to porion and from posterior nasal spine to anterior nasal spine

Facial height index (FHI), ratio of the posterior face height (distance from sella to gonion) to the anterior face height (distance from nasion to menton)

Frankfurt mandibular angle (FMA), angle between the lines from sella to nasion and from gonion to gnathion

Modified Wits Appraisal (MW), the distance between mandible 1 crown and maxilla 1 crown
